# Association Between a Novel Metabolic Score for Insulin Resistance and Mortality in People With Diabetes

**DOI:** 10.3389/fcvm.2022.895609

**Published:** 2022-05-12

**Authors:** Zhenwei Wang, Jing Xie, Junjie Wang, Wei Feng, Naifeng Liu, Yun Liu

**Affiliations:** ^1^Department of Cardiology, School of Medicine, Zhongda Hospital, Southeast University, Nanjing, China; ^2^College of Basic Medicine and Clinical Pharmacy, China Pharmaceutical University, Nanjing, China; ^3^Department of Medical Informatics, School of Biomedical Engineering and Informatics, Nanjing Medical University, Nanjing, China; ^4^Institute of Medical Informatics and Management, Nanjing Medical University, Nanjing, China

**Keywords:** diabetes, insulin resistance, metabolic score for insulin resistance, NHANES, mortality

## Abstract

**Background:**

Growing studies have shown that insulin resistance (IR) is associated with poor prognoses among patients with diabetes, whereas the association between IR and mortality has not been determined. Hence we aimed to evaluate the associations between IR and all-cause, cardiovascular diseases (CVDs) and cancer-related mortality in patients with diabetes.

**Methods:**

We enrolled 2,542 participants with diabetes with an average age of 57.12 ± 0.39 years and 52.8% men from the 1999–2014 National Health and Nutrition Examination Survey (NHANES 1999–2014). A novel metabolic score for insulin resistance (METS-IR) was considered as alternative marker of IR. Mortality data were obtained from the National Death Index records and all participants were followed up until December 31, 2015. Cox proportional hazards regression, restricted cubic spline and Kaplan-Meier survival curves were performed to evaluate the associations between METS-IR and all-cause and cause-specific mortality in patients with diabetes.

**Results:**

During 17,750 person-years of follow-up [median (months), 95% CI: 90, 87–93], 562 deaths were documented, including 133 CVDs-related deaths and 90 cancer-related deaths. Multivariate Cox regression showed that compared with Quintile 1 (METS-IR ≤ 41), METS-IR in Quintile 2, 3, and 4 was all associated with all-cause mortality (Q2 vs. Q1: HR 0.65, 95% CI 0.49–0.87, *P* = 0.004; Q3 vs. Q1: HR 0.69, 95% CI 0.50–0.96, *P* = 0.029; Q4 vs. Q1: HR 0.57, 95% CI 0.36–0.91, *P* = 0.019; respectively). Restricted cubic spline indicated that METS-IR was non-linearly associated with all-cause and CVDs-related mortality. Threshold effect analyses determined that threshold values of METS-IR for all-cause and CVDs-related mortality were both 33.33. Only METS-IR below the threshold was negatively associated with all-cause and CVDs-related mortality (HR 0.785, 95% CI 0.724–0.850, *P* < 0.001; HR 0.722, 95% CI 0.654–0.797, *P* < 0.001; respectively). Sensitivity analyses showed that when excluding participants who died within 1 years of follow-up, the results of threshold effect analyses remained consistent, whereas excluding participants with CVDs, METS-IR below the threshold was only negatively correlated with all-cause mortality. Subgroup analyses indicated that for all-cause mortality, the results were still stable in all subgroups except newly diagnosed diabetes, but for CVDs-related mortality, the association persisted only in participants who were ≤ 65 years, male, White, non-White, already diagnosed diabetes, or uesd oral drugs, insulin, insulin sensitizing drugs.

**Conclusion:**

METS-IR was non-linearly associated with all-cause and CVDs-related mortality in patients with diabetes, and METS-IR below the threshold was negatively associated with all-cause and CVDs-related mortality.

## Introduction

It is reported that diabetes is currently one of the leading indirect causes of disability and death worldwide (1), there were 425 million adults suffering from diabetes worldwide in 2017 (2), and by 2019, the number was close to 500 million (3). During this period, the number of diabetes-related deaths also increased by 5% (3). It is estimated that by 2030, the global prevalence of diabetes will reach 578 million, and the global economic burden caused by diabetes and its complications will significantly increase to $2.5 trillion (3). It can be seen that diabetes has become a global public health problem. And there is evidence that people with diabetes have a two to four times higher risks of cardiovascular diseases (CVDs) and death than those without diabetes (1). Therefore, it is very important to determine controllable factors for preventing or delaying diabetes and its complications and premature death.

Insulin resistance (IR) may be one of the main culprits that mediate the high risk of death in patients with diabetes. IR mainly refers to the decreased sensitivity of muscle, liver and adipose tissue to insulin stimulation, that is, impaired biological response, and long-term IR will lead to the dysfunction of glucose metabolism, which in turn leads to compensatory increase of insulin and hyperinsulinemia (4–6). In the longer term, IR can not only directly lead to pathological conditions such as hyperglycemia, hypertension, hyperlipidemia, hyperuricemia, obesity, thrombotic state, elevated inflammatory markers and endothelial dysfunction (7, 8), but also indirectly lead to metabolic-related diseases such as diabetes, non-alcoholic fatty liver, CVDs, and even death (9–13). At present, the associations between IR and all-cause and cause-specific mortality have been explored in some observational studies, whereas the results have been mixed. For example, several studies showed that higher levels of IR were independently associated with higher all-cause, CVDs and cancer-related mortality (14–19), whereas other studies found that the association was not statistically significant (20–23). The reason for this situation may be the heterogeneity of the study population and differences in the evaluation of IR, so it is necessary for us to use different assessment method of IR in different populations to explore the relationship between IR and all-cause and cause-specific mortality. As growing studies focus on the role of metabolism in the process of cancer, we should pay more attention to the relationship between IR and cancer-related mortality. There are currently many ways to evaluate IR. The gold standard is euglycaemic-hyperinsulinaemic clamp (EHC), whereas it is not suitable for large-scale clinical and epidemiological studies because of its invasive and expensive shortcomings (24). To this end, some researchers have developed several indexes to evaluate IR based on simple formulas, such as homeostasis model assessment for IR (HOMA-IR) (25), triglyceride to high-density lipoprotein cholesterol ratio (TG/HDL-C) and triglyceride glucose index (TyG index) (26–28). Nevertheless, these indexes ignore the role of nutritional factors such as body mass index (BMI) in IR, hence they also have certain limitations in the construction of clinical disease prediction models. In view of this, a novel non-insulin-based metabolic score of IR has been developed, namely METS-IR, which is derived from conventional clinical indexes such as fasting plasma glucose (FPG), TG, HDL-C and BMI, and has been proved to have high accuracy similar to the EHC (29). Consequently, it is an excellent tool for evaluating IR in large-scale epidemiological studies. Since METS-IR was developed, several studies have shown that it was associated with hypertension, diabetes, and coronary heart disease (29–33). Recently, our research team also found associations between METS-IR and coronary artery calcification and myocardial injury. However, to the best of our knowledge, the associations between METS-IR and all-cause and cause-specific mortality in patients with diabetes have not been reported.

Therefore, in order to fill this knowledge gap, we were aimed at prospectively exploring the associations between METS-IR and all-cause and cause-specific mortality in people with diabetes in a nationally representative sample of U.S. adults.

## Materials and Methods

### Study Population

**FIGURE 1:**
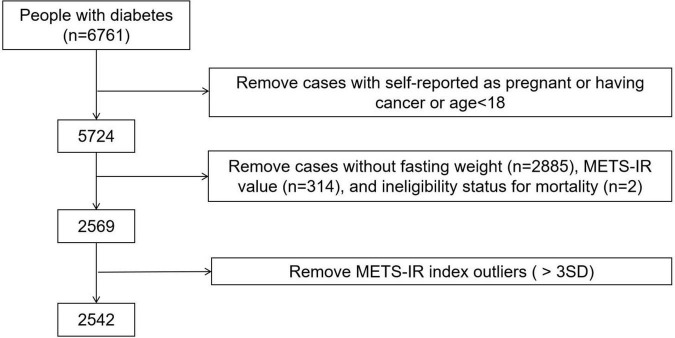
Algorithm for participant selection in the NHANES (1999–2014). Diabetes was defined as self-reported doctor diagnosis of diabetes, use of insulin or fasting glucose ≥ 7.0 mmol/L or glycated hemoglobin (HbA1c) level ≥ 6.5%, according to ADAs diabetes diagnostic criteria.

We analyzed data from the 1999 to 2014 National Health and Nutrition Examination Survey (NHANES). In the study, we only included participants with diabetes over the age of 18 because participants under the age of 18 did not have a complete medical record, we also excluded the participants who were self-reported as pregnant or having cancer or without FPG data, TG data, HDL-C data, BMI data at baseline or without follow-up data. A total 2,542 individuals were included in the final analyses, and the data cleaning algorithm was shown in Figure 1. Participants of our study have provided written informed consent, and the NHANES study project was approved by the National Center for Health Statistics of the Center for Disease Control and Prevention Institutional Review Board and in line with the Declaration of Helsinki.

### Data Collection at Baseline

The covariates included in the present study included age, sex, race/ethnicity, education level, family income, smoking status, alcohol intake, physical activity, disease status, and medication use, these data were collected from household interviews. In our study, we divided race/ethnicity into five groups: non-Hispanic White, non-Hispanic Black, Mexican American, other Hispanic, and other Races. Education level was categorized as < 9th grade, 9–11th grade, 12th grade and > 12th grade. Family income-to-poverty ratio was classified as 0–1.0, 1.0–3.0, and > 3.0. Smoking status was classified as never smoker, former smoker, and current smoker. Alcohol users were defined as those who had at least 12 drinks in the last 12 months. Ideal physical activity was defined as ≥ 150 min of moderate-intensity activities per week, ≥ 75 min of vigorous-intensity activities per week, or an equivalent combination of both. CVDs history included self-reported coronary heart disease, angina, stroke, and heart attack. According to ADAs diabetes diagnostic criteria, diabetes was defined as self-reported diagnosis, use of insulin or oral hypoglycemic medication, fasting glucose ≥ 7.0 mmol/L, or glycated hemoglobin A1c (HbA1c) ≥ 6.5% (34). Hypertension was defined as a self-reported history of hypertension. Hypercholesterolemia was defined as a self-reported history of hypercholesterolemia. We also included the BMI, blood pressure evaluated at the NHANES mobile examination centers, and total cholesterol (TC) measured in the NHANES laboratory.

### Definition of Metabolic Score for Insulin Resistance

The exposure variable of this study was METS-IR. METS-IR has been reported as a novel simple IR index, and the calculation formula was: (Ln [(2 × FPG) + TG] × BMI)/(Ln [HDL-C]), in which the blood indicators were derived from the venous blood of participants who fasted for more than 8 h (29).

### Ascertainment of Mortality

The outcome variables including all-cause, CVDs and cancer-related mortality were defined based on ICD10 code. These mortality data were obtained from the National Center for Health Statistics by using probabilistic record matching with death certificate data found in the National Death Index (NCHS Linked Mortality File) by December 31, 2015. Follow-up time was defined as the period between the NHANES examination date and the last known date about each person living or death (35).

### Statistical Analysis

Since the NHANES is a complex multi-stage probabilistic sampling design, we used the weights of the fasting subsample and adjusted the weights to account for multiple cycles. Participants were ranked in the METS-IR quintile: Quintile 1 (≤ 41), Quintile 2 (41–47), Quintile 3 (47–54), Quintile 4 (54–63), Quintile 5 (> 63). Baseline characteristics were presented as mean ± SE or proportions. Either a weighted chi-square test (categorical variables) or a weighted linear regression model (continuous variables) was used to calculate differences between different METS-IR groups (36).

Multivariate Cox proportional hazard models were used to obtain the hazard ratios (HRs) to assess the risks of all-cause, CVDs and cancer-related mortality. In the multivariate models, we adjusted for age, gender and race/ethnicity in model 1. In model 2, we further adjusted for BMI, education level, family income-poverty ratio, alcohol user, smoking status, ideal physical activity, and daily calorie intake. In model 3, we further adjusted for duration of diabetes, hypoglycemic drugs, lipid-lowering drugs, self-reported hypertension, hypercholesterolemia, and CVDs, systolic blood pressure (SBP), and TC. Missing data of covariates were handled by the Multiple imputation. The linear trend was tested by assigning a median value for each group as a continuous variable (37).

Restricted cubic spline models were used to detect the non-linear relationship between METS-IR index and mortality. If non-linear relationships were found, two-piecewise linear regression models would be used to illustrate how the relationships differ by threshold point. The threshold value was estimated by trying all possible values and choosing the threshold value with highest likelihood. Logarithmic likelihood ratio test was employed to compare the one-line linear regression model with a two-piecewise linear model.

Stratified analyses were also conducted by age (≤ 65 or > 65 years), gender (male or female), race/ethnicity (White or non-White), stages of diabetes (newly diagnosed or already diagnosed), oral drugs (yes or no), insulin drugs (yes or no), insulin sensitizing drugs (yes or no), non-insulin sensitizing drugs (yes or no). In the sensitivity analyses, we excluded those who died within the first year of follow-up or had CVDs at baseline. A two-tailed *P*-value < 0.05 was defined as statistically significant. All statistical analyses were performed in R software (4.1.0) and the survey package (version 4.1-1) in R to account for the complex sampling design.

## Results

### Baseline Characteristics by Quintile of Metabolic Score for Insulin Resistance

A total of 2,542 individuals with an average age of 57.12 ± 0.39 years and 52.8% men were enrolled in our study, and their baseline characteristics grouped according to the quintile of METS-IR were shown in Table 1. The participants with higher METS-IR were younger, more likely to be non-Hispanic White, more likely to suffer from hypertension, and had a shorter history of diabetes, more use of hypoglycemic drugs and higher BMI than those with lower METS-IR. More importantly, participants with higher METS-IR had lower all-cause mortality (*P* < 0.001).

**TABLE 1:** Baseline characteristics of participants with diabetes by quintile of METS-IR in NHANES 1999–2014.

	Total	Quintile 1 (≤ 41)	Quintile 2 (41–47)	Quintile 3 (47–54)	Quintile 4 (54–63)	Quintile 5 (> 63)	*P*-value
METS-IR	52.44 ± 0.47	35.74 ± 0.25	44.40 ± 0.10	50.74 ± 0.14	58.6 ± 0.19	72.62 ± 0.60	<0.001
Age (mean ± SE), years	57.12 ± 0.39	60.29 ± 0.95	59.79 ± 0.82	58.18 ± 0.89	56.31 ± 0.84	51.03 ± 0.85	<0.001
Gender							0.708
Male	1341 (52.8)	268 (52.8)	266 (52.5)	288 (56.6)	262 (51.5)	257 (50.4)	
Female	1201 (47.2)	239 (47.2)	241 (47.5)	221 (43.4)	221 (48.5)	252 (49.6)	
Race/ethnicity							<0.001
Mexican American	256 (10.1)	29 (5.8)	55 (10.9)	59 (11.5)	59 (11.5)	54 (10.6)	
Other Hispanic	165 (6.5)	42 (8.3)	34 (6.8)	43 (8.5)	28 (5.5)	18 (3.5)	
Non-Hispanic White	1505 (59.2)	270 (53.2)	279 (55.0)	311 (61.0)	318 (62.5)	326 (64.2)	
Non-Hispanic black	402 (15.8)	82 (16.2)	89 (17.6)	71 (14.0)	81 (15.8)	79 (15.5)	
Other races	214 (8.4)	84 (16.5)	50 (9.8)	25 (5.0)	23 (4.6)	32 (6.2)	
BMI (mean ± SE), kg/m^2^	32.50 ± 0.22	24.42 ± 0.15	28.70 ± 0.14	31.71 ± 0.19	35.82 ± 0.25	41.84 ± 0.44	<0.001
SBP (mean ± SE), mmHg	130.14 ± 0.56	132.11 ± 1.55	129.51 ± 1.12	130.71 ± 1.38	129.36 ± 1.10	129.02 ± 1.34	0.141
Total cholesterol (mean ± SE), mg/dL	192.00 ± 1.32	189.77 ± 2.80	192.02 ± 2.52	190.70 ± 3.08	193.41 ± 2.86	194.12 ± 3.91	0.279
Alcohol user							0.341
Yes	1712 (67.3)	336 (66.2)	335 (66.1)	358 (70.2)	362 (71.0)	322 (63.2)	
No	830 (32.7)	172 (33.8)	172 (33.9)	152 (29.8)	148 (29.0)	187 (36.8)	
Smoking status							0.132
Never smoker	1255 (49.4)	250 (49.2)	253 (50.0)	219 (43.0)	246 (48.4)	286 (56.3)	
Ever smoker	848 (33.3)	165 (32.5)	185 (36.6)	188 (36.9)	167 (32.8)	143 (28.0)	
Current smoker	439 (17.3)	93 (18.3)	68 (13.4)	102 (20.1)	96 (18.8)	80 (15.7)	
Education levels							0.221
<9th grade	306 (12.0)	56 (11.0)	82 (16.1)	77 (15.0)	53 (10.5)	38 (7.5)	
9–11th grade	403 (15.9)	81 (15.9)	73 (14.3)	84 (16.5)	84 (16.4)	83 (16.2)	
12th grade	674 (26.5)	125 (24.6)	133 (26.2)	139 (27.3)	134 (26.3)	144 (28.2)	
>12th grade	1158 (45.6)	246 (48.4)	220 (43.4)	210 (41.2)	238 (46.8)	244 (48.0)	
Family income-poverty ratio							0.996
≤1.0	466 (18.3)	98 (19.3)	92 (18.1)	87 (17.2)	97 (19.1)	91 (18.0)	
1.0–3.0	1118 (44.0)	220 (43.3)	232 (45.7)	222 (43.7)	218 (42.9)	225 (44.3)	
>3.0	958 (37.7)	190 (37.4)	183 (36.2)	200 (39.2)	194 (38.0)	192 (37.7)	
Ideal physical activity							0.056
Yes	996 (39.2)	224 (44.2)	218 (43.0)	203 (39.9)	180 (35.4)	170 (33.4)	
No	1546 (60.8)	283 (55.8)	289 (57.0)	306 (60.1)	329 (64.6)	339 (66.6)	
Duration of diabetes							0.007
≤3 years	840 (33.0)	121 (23.9)	151 (29.8)	170 (33.4)	191 (37.6)	207 (40.6)	
3–10 years	909 (35.8)	198 (39.1)	178 (35.2)	196 (38.5)	170 (33.3)	166 (32.7)	
>10 years	793 (31.2)	188 (37.0)	177 (35.0)	143 (28.1)	148 (29.1)	136 (26.7)	
Hypoglycemic drugs							0.001
No insulin or oral drugs	1047 (41.2)	198 (39.0)	209 (41.2)	206 (40.5)	235 (46.1)	199 (39.2)	
Only oral drugs	1116 (43.9)	215 (42.4)	235 (46.3)	242 (47.4)	216 (42.5)	208 (40.9)	
Only insulin	196 (7.7)	63 (12.5)	44 (8.6)	34 (6.7)	24 (4.6)	32 (6.2)	
Oral drugs and insulin	183 (7.2)	31 (6.1)	20 (3.9)	28 (5.4)	35 (6.8)	70 (13.7)	
Lipid-lowering drugs							0.651
Yes	1017 (40.0)	210 (40.9)	183 (36.2)	216 (42.6)	211 (41.5)	197 (38.8)	
No	1525 (60.0)	303 (59.1)	323 (63.8)	291 (57.4)	298 (58.5)	311 (61.2)	
Hypertension							<0.001
Yes	1523 (59.9)	232 (45.7)	285 (56.2)	319 (62.7)	332 (65.3)	354 (69.6)	
No	1019 (40.1)	275 (54.3)	222 (43.8)	190 (37.3)	177 (34.7)	154 (30.4)	
Hypercholesterolemia							0.949
Yes	1486 (58.4)	294 (58.0)	290 (57.3)	308 (60.5)	301 (59.0)	292 (57.4)	
No	1056 (41.6)	213 (42.0)	217 (42.7)	201 (39.5)	209 (41.0)	217 (42.6)	
CVDs							0.402
Yes	559 (22.0)	97 (19.0)	113 (22.3)	120 (23.6)	129 (25.3)	101 (19.8)	
No	1983 (78.0)	411 (81.0)	394 (77.7)	389 (76.4)	381 (74.7)	408 (80.2)	
**Outcomes**							
All-cause mortality							<0.001
Yes	468 (18.4)	134 (26.5)	101 (19.9)	94 (18.4)	74 (14.6)	64 (12.7)	
No	2074 (81.6)	373 (73.5)	406 (80.1)	416 (81.6)	435 (85.4)	444 (87.3)	
CVDs-related mortality							0.508
Yes	116 (4.5)	30 (5.9)	27 (5.4)	21 (4.1)	18 (3.6)	19 (3.7)	
No	2426 (95.5)	477 (94.1)	480 (94.6)	488 (95.9)	491 (96.4)	490 (96.3)	
Cancer-related mortality							0.357
Yes	68 (2.7)	12 (2.4)	17 (3.3)	19 (3.8)	13 (2.5)	7 (1.3)	
No	2474 (97.3)	495 (97.6)	490 (96.7)	490 (96.2)	497 (97.5)	502 (98.7)	

*Data were numbers (percentages) unless otherwise indicated. All estimates accounted for complex survey designs.*

### Associations Between Metabolic Score for Insulin Resistance and All-Cause and Cause-Specific Mortality

During 17,750 person-years of follow-up [median (month), 95% CI: 90, 87–93], 562 deaths (all-cause) were documented, including 133 CVDs-related deaths and 90 cancer-related deaths. Multivariate Cox proportional hazard regression analyses results of Table 2 showed that METS-IR, as a continuous variable, was not associated with all-cause, CVDs, and cancer-related mortality after adjusting for age, sex, race/ethnicity, education level, family income-poverty ratio, alcohol user, smoking status, ideal physical activity, duration of diabetes, hypoglycemic drugs, lipid-lowering drugs, self-reported hypertension, hypercholesterolemia, CVDs, SBP, and TC. When as a classified variable, compared with Quintile 1 (METS-IR ≤ 41), METS-IR in Quintile 2, Quintile 3, and Quintile 4 was all independently associated with all-cause mortality (Q2 vs. Q1: HR 0.65, 95% CI 0.49–0.87, *P* = 0.004; Q3 vs. Q1: HR 0.69, 95% CI 0.50–0.96, *P* = 0.029; Q4 vs. Q1: HR 0.57, 95% CI 0.36–0.91, *P* = 0.019; respectively).

**TABLE 2:** HRs (95% CIs) for all-cause and cause-specific mortality among participants with diabetes in NHANES 1999–2014.

	Mortality rate (per 1,000 person-years)	Model 1	Model 2	Model 3
**All-cause mortality**				
MEST-IR index (per 1 unit increment)	25.54	0.99 (0.98, 1.00), 0.227	0.99 (0.98, 1.00), 0.230	0.99 (0.98, 1.00), 0.111
**MEST-IR index group**				
Quintile 1 (≤ 41)	38.80	1.00	1.00	1.00
Quintile 2 (41–47)	27.82	0.70 (0.52, 0.94), 0.019	0.72 (0.53, 0.96), 0.028	0.65 (0.49, 0.87), 0.004
Quintile 3 (47–54)	24.78	0.69 (0.50, 0.96), 0.026	0.70 (0.51, 0.96), 0.027	0.69 (0.50, 0.96), 0.029
Quintile 4 (54–63)	19.60	0.65 (0.41, 1.02), 0.062	0.64 (0.41, 1.01), 0.056	0.57 (0.36, 0.91), 0.019
Quintile 5 (> 63)	17.66	0.87 (0.58, 1.31), 0.510	0.90 (0.61, 1.33), 0.612	0.81 (0.55, 1.21), 0.306
P trend		0.335	0.327	0.171
**CVDs-related mortality**				
MEST-IR index (per 1 unit increment)	6.31	1.00 (0.97, 1.02), 0.710	1.00 (0.97, 1.02), 0.780	0.99 (0.97, 1.01), 0.358
**MEST-IR index group**				
Quintile 1 (≤ 41)	8.70	1.00	1.00	1.00
Quintile 2 (41–47)	7.56	0.81 (0.44, 1.50), 0.504	0.80 (0.42, 1.50), 0.480	0.71 (0.38, 1.35), 0.300
Quintile 3 (47–54)	5.55	0.65 (0.34, 1.25), 0.198	0.64 (0.33, 1.26), 0.199	0.58 (0.28, 1.22), 0.151
Quintile 4 (54–63)	4.78	0.72 (0.35, 1.51), 0.389	0.71 (0.33, 1.55), 0.395	0.58 (0.26, 1.30), 0.184
Quintile 5 (> 63)	5.17	1.24 (0.64, 2.37), 0.526	1.33 (0.69, 2.54), 0.394	1.04 (0.51, 2.13), 0.915
P trend		0.824	0.756	0.811
**Cancer-related mortality**				
MEST-IR index (per 1 unit increment)	3.70	1.00 (0.97, 1.02), 0.938	1.00 (0.97, 1.02), 0.865	0.99 (0.97, 1.02), 0.721
**MEST-IR index group**				
Quintile 1 (≤ 41)	3.53	1.00	1.00	1.00
Quintile 2 (41–47)	4.58	1.36 (0.64, 2.90), 0.430	1.41 (0.68, 2.94), 0.359	1.44 (0.71, 2.93), 0.309
Quintile 3 (47–54)	5.14	1.72 (0.75, 3.96), 0.203	1.75 (0.74, 4.16), 0.203	1.73 (0.71, 4.20), 0.226
Quintile 4 (54–63)	3.35	1.36 (0.65, 2.81), 0.412	1.32 (0.66, 2.65), 0.432	1.30 (0.60, 2.80), 0.509
Quintile 5 (> 63)	1.84	1.14 (0.30, 4.35), 0.846	1.11 (0.30, 4.03), 0.877	1.05 (0.26, 4.33), 0.942
P trend		0.756	0.812	0.935

*Model 1: adjusted for age, sex and race/ethnicity.*

*Model 2: further adjusted (from Model 1) for education level, family income-poverty ratio, alcohol user, smoking status, and ideal physical activity.*

*Model 3: further adjusted (from Model 2) for duration of diabetes, hypoglycemic drugs, lipid-lowering drugs, self-reported hypertension, hypercholesterolemia, and CVDs, systolic blood pressure, and total cholesterol.*

**FIGURE 2:**
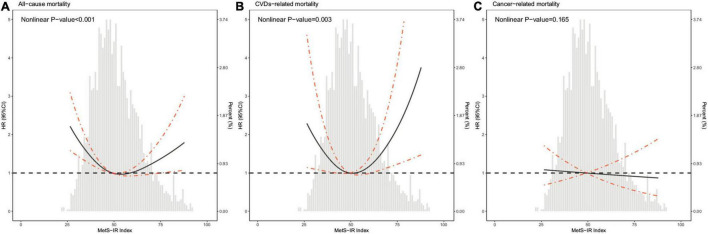
Hazard ratios for all-cause mortality **(A)**, CVDs-related mortality **(B)**, and cancer-related mortality **(C)** according to METS-IR index and the histogram of probability distribution were presented in the background. Hazard ratios were calculated by Cox models after adjusting for age, sex, race, education level, family income-poverty ratio, alcohol user, smoking status, ideal physical activity, duration of diabetes, hypoglycemic drugs, lipid-lowering drugs, self-reported hypertension, hypercholesterolemia, and CVDs, systolic blood pressure, and total cholesterol.

As shown in Figure 2, the restricted cubic spline with adjustment for model 3 showed that METS-IR was non-linearly associated with risks of all-cause and CVDs-related deaths (all non-linear *P* < 0.05). The threshold effect analyses in Table 3 indicated that the threshold effect points of METS-IR for all-cause and CVDs-related mortality were both 33.33. At the right side of the threshold point (METS-IR ≥ 33.33), risks of all-cause and CVDs-related deaths rose with the increase of METS-IR, while this association was not statistically significant. Nevertheless, at the left side of the threshold point (METS-IR < 33.33), METS-IR was significantly negatively associated with risks of all-cause and CVDs-related deaths (HR 0.785, 95% CI 0.724–0.850, *P* < 0.001; HR 0.722, 95% CI 0.654–0.797, *P* < 0.001; respectively). And we found that the 2-piecewise multivariate Cox proportional hazard regression model was superior to the l-line model for fitting the associations between METS-IR and all-cause and CVDs-related mortality (P for log likelihood ratio test < 0.001). Sensitivity analysis showed that when participants who died within 1 year of follow-up were excluded, the results were still consistent with the main results of Table 3, that is, when METS-IR < threshold value of 33.33, it was still significantly negatively associated with risks of all-cause and CVDs-related deaths, whereas excluding participants with CVDs, METS-IR below the threshold was only negatively correlated with all-cause mortality (Tables 4, 5).

**TABLE 3:** Threshold effect analysis of METS-IR on all-cause and CVDs-related mortality among diabetes.

	All-cause mortality	CVDs-related mortality
Threshold value	33.33	33.33
<Threshold value	0.785 (0.724, 0.850), <0.001	0.722 (0.654, 0.797), <0.001
≥Threshold value	1.001 (0.989, 1.013), 0.932	1.007 (0.983, 1.031), 0.587
P for log likelihood ratio test	<0.001	<0.001

*Data were presented as hazard ratios, 95% confidence intervals, and P-value. The two-piecewise linear regression models were adjusted for age, sex, race, education level, family income-poverty ratio, alcohol user, smoking status, ideal physical activity, duration of diabetes, hypoglycemic drugs, lipid-lowering drugs, self-reported hypertension, hypercholesterolemia, and CVDs, systolic blood pressure, and total cholesterol.*

**TABLE 4:** Threshold effect analyses of METS-IR on all-cause and CVDs-related mortality among diabetes after excluding participants who died within 1 years of follow-up (*n* = 2,372).

	All-cause mortality	CVDs-related mortality
Threshold value	33.33	33.33
<Threshold value	0.782 (0.731, 0.837), <0.001	0.722 (0.641, 0.814), <0.001
≥Threshold value	1.001 (0.986, 1.015), 0.935	1.003 (0.97, 1.036), 0.866
P for log likelihood ratio test	<0.001	<0.001

*Data were presented as hazard ratios, 95% confidence intervals, and P-value. The two-piecewise linear regression models were adjusted for age, sex, race, education level, family income-poverty ratio, alcohol user, smoking status, ideal physical activity, duration of diabetes, hypoglycemic drugs, lipid-lowering drugs, self-reported hypertension, hypercholesterolemia, and CVDs, systolic blood pressure, and total cholesterol.*

**TABLE 5:** Threshold effect analyses of METS-IR on all-cause and CVDs-related mortality among diabetes after excluding participants who had CVDs at baseline (*n* = 1,943).

	All-cause mortality	CVDs-related mortality
Threshold value	33.09	33.09
<Threshold value	0.810 (0.720, 0.910), <0.001	0.873 (0.568, 1.342), 0.536
≥Threshold value	1.003 (0.986, 1.020), 0.754	0.993 (0.948, 1.039), 0.755
P for log likelihood ratio test	0.002	0.458

*Data were presented as hazard ratios, 95% confidence intervals, and P-value. The two-piecewise linear regression models were adjusted for age, sex, race, education level, family income-poverty ratio, alcohol user, smoking status, ideal physical activity, duration of diabetes, hypoglycemic drugs, lipid-lowering drugs, self-reported hypertension, hypercholesterolemia, systolic blood pressure, and total cholesterol.*

**FIGURE 3:**
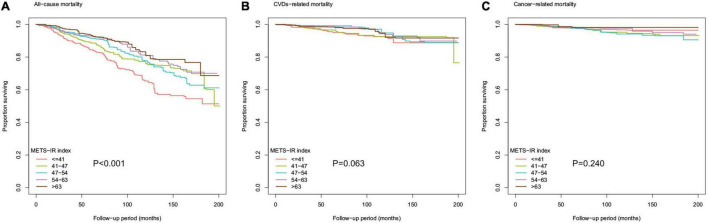
Kaplan-Meier survival curve for **(A)** all-cause mortality, **(B)** CVDs-related mortality, and **(C)** cancer-related mortality by METS-IR index.

In addition, as shown in Figure 3, the Kaplan-Meier survival curve stratified according to the quintile of METS-IR demonstrated that the cumulative incidence of all-cause death decreased with the increase of METS-IR (log-rank test, *P* < 0.001).

### Subgroup Analyses

Table 6 showed the results of stratified analyses of sixteen subgroups stratified by age, sex, race/ethnicity, diabetes and hypoglycemic drugs. With the exception of newly diagnosed diabetes subgroup, regardless of the threshold of METS-IR, METS-IR below the threshold was independently negatively associated with risk of all-cause death in other subgroups. However, METS-IR less than the threshold was only negatively associated with CVDs-related death risk in subgroups with ≤ 65 years, male, White, non-White, already diagnosed diabetes, or uesd oral drugs, insulin, insulin sensitizing drugs.

**TABLE 6:** Stratification analysis of METS-IR index with all-cause and CVDs-related mortality.

		All-cause mortality	CVDs-related mortality
**Age (years)**			
≤65 (*n* = 1,564)	Threshold value	34.34	34.34
	<Threshold value	0.814 (0.741, 0.893), <0.001	0.678 (0.588, 0.781), <0.001
	≥Threshold value	1.004 (0.983, 1.025), 0.710	1.015 (0.970, 1.062), 0.530
	P for log likelihood ratio test	<0.001	<0.001
>65 (*n* = 978)	Threshold value	32.22	32.22
	<Threshold value	0.770 (0.698, 0.850),<0.001	0.845 (0.514, 1.390), 0.507
	≥ Threshold value	0.996 (0.981, 1.011), 0.610	0.996 (0.957, 1.035), 0.824
	P for log likelihood ratio test	<0.001	0.220
**Gender**			
Male (*n* = 1,352)	Threshold value	34.3	34.3
	< Threshold value	0.773 (0.722, 0.827),<0.001	0.725 (0.638, 0.825),<0.001
	≥ Threshold value	1.011 (0.996, 1.026), 0.153	1.012 (0.976, 1.050), 0.522
	P for log likelihood ratio test	<0.001	<0.001
Female (*n* = 1,190)	Threshold value	32.44	32.44
	< Threshold value	0.811 (0.711, 0.926), 0.002	0.856 (0.646, 1.134), 0.278
	≥ Threshold value	0.983 (0.961, 1.006), 0.145	1.002 (0.939, 1.069), 0.956
	P for log likelihood ratio test	0.005	0.259
**Race/ethnicity**			
White (*n* = 898)	Threshold value	33.64	33.64
	< Threshold value	0.795 (0.709, 0.891), < 0.001	0.738 (0.575, 0.947), 0.017
	≥ Threshold value	1.001 (0.976, 1.026), 0.957	1.007 (0.956, 1.060), 0.806
	P for log likelihood ratio test	0.002	0.023
Non-White (*n* = 1,644)	Threshold value	33.38	33.38
	<Threshold value	0.778 (0.678, 0.894), < 0.001	0.694 (0.624, 0.771), <0.001
	≥ Threshold value	0.998 (0.98, 1.019), 0.844	1.005 (0.967, 1.045), 0.788
	P for log likelihood ratio test	<0.001	<0.001
**Stages of diabetes**			
Newly diagnosed (*n* = 742)	Threshold value	33.34	33.34
	< Threshold value	0.902 (0.796, 1.022), 0.106	0.953 (0.284, 3.198), 0.938
	≥ Threshold value	0.994 (0.966, 1.022), 0.660	1.023 (0.961, 1.089), 0.476
	P for log likelihood ratio test	0.268	0.825
Already diagnosed (*n* = 1,800)	Threshold value	33.38	33.38
	< Threshold value	0.761 (0.692, 0.838), <0.001	0.688 (0.605, 0.783), <0.001
	≥ Threshold value	1.001 (0.987, 1.015), 0.859	1.002 (0.971, 1.034), 0.920
	P for log likelihood ratio test	<0.001	<0.001
**Hypoglycemic drugs**			
**Oral drugs**			
Yes (*n* = 1,311)	Threshold value	34.30	34.30
	< Threshold value	0.740 (0.693, 0.789), <0.001	0.711 (0.627, 0.805), <0.001
	≥ Threshold value	1.011 (0.995, 1.028), 0.182	1.006 (0.965, 1.049), 0.769
	P for log likelihood ratio test	<0.001	<0.001
No (*n* = 1,231)	Threshold value	32.37	32.37
	< Threshold value	0.819 (0.710, 0.945), 0.006	0.742 (0.564, 0.975), 0.032
	≥ Threshold value	0.992 (0.972, 1.012), 0.413	1.013 (0.960, 1.069), 0.638
	P for log likelihood ratio test	0.019	0.130
**Insulin**			
Yes (*n* = 455)	Threshold value	32.37	32.37
	< Threshold value	0.686 (0.567, 0.830), < 0.001	0.490 (0.303, 0.791), 0.004
	≥ Threshold value	0.982 (0.951, 1.014), 0.276	0.973 (0.894, 1.060), 0.535
	P for log likelihood ratio test	<0.001	<0.001
No (*n* = 2,087)	Threshold value	33.38	33.38
	< Threshold value	0.813 (0.719, 0.919), 0.001	0.811 (0.482, 1.363), 0.428
	≥ Threshold value	1.005 (0.990, 1.020), 0.553	1.016 (0.983, 1.051), 0.336
	P for log likelihood ratio test	<0.001	0.052
**Insulin sensitizing drugs**			
Yes (*n* = 994)	Threshold value	33.75	33.75
	< Threshold value	0.673 (0.611, 0.742), <0.001	0.657 (0.544, 0.794), <0.001
	≥ Threshold value	1.017 (1.000, 1.035), 0.049	1.013 (0.982, 1.044), 0.420
	P for log likelihood ratio test	<0.001	<0.001
No (*n* = 1,360)	Threshold value	32.37	32.37
	< Threshold value	0.787 (0.666, 0.930), 0.005	0.646 (0.533, 0.784), <0.001
	≥ Threshold value	0.994 (0.971, 1.018), 0.637	1.006 (0.950, 1.065), 0.847
	P for log likelihood ratio test	<0.001	<0.001
**Non-insulin sensitizing drugs**			
Yes (*n* = 658)	Threshold value	34.94	34.94
	< Threshold value	0.733 (0.658, 0.816), <0.001	0.794 (0.530, 1.189), 0.262
	≥ Threshold value	1.015 (0.994, 1.036), 0.158	0.992 (0.929, 1.060), 0.813
	P for log likelihood ratio test	<0.001	0.156
No (*n* = 1,884)	Threshold value	32.68	32.68
	<Threshold value	0.799 (0.706, 0.903), <0.001	0.682 (0.599, 0.775), <0.001
	≥ Threshold value	0.996 (0.982, 1.011), 0.623	1.015 (0.980, 1.051), 0.412
	P for log likelihood ratio test	<0.001	<0.001

*All the model adjusted for age, sex, race/ethnicity, BMI, education level, family income-poverty ratio, alcohol user, smoking status, ideal physical activity, daily calorie intake, duration of diabetes, hypoglycemic drugs, lipid-lowering drugs, self-reported hypertension, hypercholesterolemia, and CVDs, systolic blood pressure, and total cholesterol, with exception of stratifying factors.*

## Discussion

For all we know, our study was the first report on the associations between METS-IR and all-cause and cause-specific mortality in patients with diabetes, and the first to find that METS-IR was negatively associated with risks of all-cause and CVDs-related deaths in a certain range. That is, after adjusting for confounding factors, METS-IR less than the threshold point was significantly negatively associated with the risk of all-cause or CVDs-related death.

IR is mainly an acquired disease, which is related to overnutrition and metabolic abnormalities. It exists more or less in patients with diabetes, which is usually accompanied by hyperinsulinemia and hyperglycemia, and it is also considered to be an important risk factor for cardiovascular and metabolic related diseases (38). Therefore, compared with the general population, people with IR or diabetes generally have a shorter life expectancy and higher all-cause and CVDs-related mortality. However, although genetic causes have been found, the clinical definition of IR is still difficult to determine because of lack of generally accepted method to evaluate IR, which also creates a great challenge to the application of IR in epidemiological studies (39, 40). In recent decades, HOMA-IR, TG/HDL-C, and TyG are often used as markers of IR in epidemiological studies, whereas in recent years, METS-IR, an alternative indicator that is considered to fully represent IR, has gradually attracted attention. Perhaps due to different evaluation methods of IR, there are no consensus on the relationships between IR represented by the above indexes and all-cause and cause-specific mortality. For example, in a large prospective study involving 5,511 adults without diabetes, Ausk et al. found that after adjusting for potential confounding factors, higher HOMA-IR was independently associated with higher risks of all-cause and CVDs-related deaths, but not with the risk of cancer death (41). Another study showed that among obese participants, HOMA-IR was negatively associated with total and CVDs-related mortality, while among lean participants, individuals with higher HOMA-IR had about twice the risk of total or CVDs-related death as those with lower HOMA-IR (22). Additionally, Pan et al. demonstrated that higher HOMA-IR was independently associated with higher all-cause and cancer-related mortality in a cohort study of 22,837 postmenopausal women, and sensitivity analysis showed that diabetes failed to affect findings (18). Furthermore, two studies have shown that TyG was non-linearly associated with all-cause and CVDs-related mortality, they found that there was a threshold effect between TyG and risk of death, when TyG was less than the threshold point, similar to our study, it was also negatively correlated with all-cause mortality., whereas when TyG was greater than the threshold point, its harmful effect on death risk increased gradually (42, 43). Nevertheless, surprisingly, in a study including 50,673 hemodialysis patients, Chang et al. indicated that the 10th decile group (reference: sixth decile of TG/HDL-C) could reduce the risk of all-cause death and CVDs-related death by 14 and 23%, respectively, and these associations remained remarkably consistent and significant in different subgroups (44). Besides, a cohort study with 4,742 older people free of diabetes showed that higher HOMA-IR was not associated with all-cause mortality or CVDs-related mortality, and the results were similar when fasting insulin was considered as an exposure alone (20). However, our study also found that IR represented by METS-IR had a non-linear association with the risk of all-cause or CVDs-related death, and threshold effect analysis showed that only the association on the left side of the threshold point was statistically significant, that is, a significant negative association.

To sum up, we found that the association between IR reflected by different indicators and mortality was inconsistent, and the reason for this contradiction might be the difference in the evaluation methods of IR and the heterogeneity of the study population, or it may be caused by unknown mechanism.

Most cells need to rely on insulin for glucose uptake. However, due to the disorder and destruction of various molecular pathways, the sensitivity of tissues to insulin signals is reduced, which leads to IR. IR is the pathological basis of many metabolism-related diseases, although its exact cause is not completely clear, there has been some evidences that inflammation, mitochondrial dysfunction, oxidative stress, endoplasmic reticulum stress and insulin receptor mutation may be the molecular mechanisms involved in IR (8, 45–47). Generally speaking, it is generally accepted that these mechanisms are used to explain the risk of IR, while our findings and other studies unexpectedly showed that METS-IR within a specific range was negatively associated with all-cause mortality or CVDs-related mortality. Nonetheless, this did not mean that the findings were not credible. For instance, there are several evidences that the impaired insulin signal can appropriately prolong the life expectancy of caenorhabditis elegans, flies, worms and mice, among which the mutation of insulin receptor gene can enhance the resistance to oxidative stress and aging (48–50). Given the well-known harmful effects of IR, the findings have been questioned whether they are applicable to human beings. However, previous study have shown that gene mutations in the insulin/insulin-like growth factor-1 signaling pathway, such as FOXO3A, failed to generate a negative effect on life expectancy of different ethnic populations (51). In addition, there are some epidemiological evidences to support the unexpected finding, that is, they found that IR represented by HOMA-IR, TyG, and TG/HDL-C was significantly negatively correlated with all-cause mortality or CVDs-related mortality in a certain range (22, 43, 44). From the perspective of reverse thinking, lower IR, or IR within a specific range may be a potential mechanism against metabolic disorders, thereby enhancing cellular defense (52, 53). Nevertheless, IR is affected by many factors, among which obesity plays the most important role. In obese individuals, IR may act as a compensatory adaptation to limit glucose absorption by cells, and thus play a certain compensatory role in the life expectancy of obese patients (54). Similarly, we used an index containing BMI that can reflect obesity as a marker of IR, and found that there was a non-linear association between METS-IR and mortality. METS-IR below the threshold point was negatively associated with all-cause and CVDs-related mortality, while METS-IR above the threshold point had no significant effect on mortality. In other words, individuals below the threshold point represented thin people, and the role of IR in thin people was weakened, so individuals with normal or good metabolism had a lower risk of death. Contrarily, individuals above the threshold point represented obese people, the role of IR in obese people was enhanced, which was supposed to increase the risk of death, whereas the subsequent protective adaptation to IR, cellular defense system and reflex resistance to oxidative stress could counteract the harmful effects of IR on death, which further explained why the effect of METS-IR above the threshold point on mortality was not statistically significant. Nevertheless, additional studies are warranted to further explore the underlying mechanisms of association between METS-IR and mortality.

## Study Limitations

Although our study had the advantages of large sample size, prospective study design, comprehensive data and strong representativeness of the study population, there were still several limitations. Firstly, the causal association between METS-IR and mortality was unclear in this observational study. Secondly, we only evaluated the associations between METS-IR and all-cause and cause-specific mortality in people with diabetes, but not in other populations. Thirdly, although there were some evidences that METS-IR could be used as an alternative marker of IR, we failed to assess the effects of other IR markers on mortality in same population. Fourthly, self-reported CVDs can be misdiagnosis of CVDs. Fifthly, our study did not further classify diabetes into type 1 and type 2. Sixthly, increasing scholars begin to pay attention to the role of metabolism in the occurrence and development of cancer, and we also tried to explore the relationship between IR and cancer-related mortality. Although previous studies have shown that IR and metabolic syndrome were associated with higher risks of cancer and cancer-related death (55, 56), our study failed to find a linear or non-linear relationship between METS-IR and cancer-related mortality, which may be due to the heterogeneity of the study population and the small sample size. Finally, there may be other residual confounding factors that have not been controlled, such as oxidative stress, inflammatory, thrombotic status, and genetic susceptibility.

## Conclusion

In conclusion, we confirmed that METS-IR, as a novel alternative marker of IR, had a non-linear association with all-cause and CVDs-related mortality in patients with diabetes, and METS-IR within a specific range was negatively associated with all-cause and CVDs-related mortality, which also highlighted the importance of developing different management strategies based on different populations and degrees of IR in preventing premature death in patients with diabetes.

## Data Availability Statement

The original contributions presented in the study were included in the article/supplementary material, further inquiries can be directed to the corresponding author/s.

## Ethics Statement

The studies involving human participants were reviewed and approved by the National Center for Health Statistics of the Center for Disease Control and Prevention Institutional Review Board. The patients/participants provided their written informed consent to participate in this study.

## Author Contributions

ZW and JX conducted analyses and wrote the first draft of the article. JW and WF collected and assembled the data. NL and YL conceived of the study design. All authors contributed to the interpretation of the results and critical revision of the manuscript for important intellectual content, read, and approved the final version of the manuscript.

## Conflict of Interest

The authors declare that the research was conducted in the absence of any commercial or financial relationships that could be construed as a potential conflict of interest.

## Publisher’s Note

All claims expressed in this article are solely those of the authors and do not necessarily represent those of their affiliated organizations, or those of the publisher, the editors and the reviewers. Any product that may be evaluated in this article, or claim that may be made by its manufacturer, is not guaranteed or endorsed by the publisher.

## Abbreviations

IR: Insulin resistance
NHANES: National Health and Nutrition Examination Survey
METS-IR: Metabolic score for insulin resistance
CVDs: Cardiovascular diseases
EHC: Euglycaemic-hyperinsulinaemic clamp
HOMA-IR: Homeostasis model assessment for IR
TG/HDL-C: Triglyceride to high-density lipoprotein cholesterol ratio
TyG index: Triglyceride glucose index
BMI: Body mass index
FPG: Fasting plasma glucose
HbA1c: Glycated hemoglobin A1c
TC: Total cholesterol
SBP: Systolic blood pressure
HR: Hazard ratio
CI: Confidence interval.
